# Sézary syndrome presenting as vitiligo-like leukoderma with response and repigmentation to mogamulizumab and extracorporeal photopheresis

**DOI:** 10.1016/j.jdcr.2025.10.072

**Published:** 2025-11-24

**Authors:** Elizabeth Anderson, Jordyn Puccio, Eric Mou, Brian Swick, Vincent Liu

**Affiliations:** a Department of Dermatology, University of Iowa Hospitals and Clinics, Iowa City, Iowa; b Department of Internal Medicine, Trinity Health Livonia Hospital, Livonia, Michigan; c Division of Hematology, Oncology, and Blood & Marrow Transplant, University of Iowa Hospitals and Clinics, Iowa City, Iowa; d Department of Pathology, University of Iowa Hospitals and Clinics, Iowa City, Iowa

**Keywords:** cutaneous T-cell lymphoma, extracorporeal photopheresis, mogamulizumab, Sézary syndrome, vitiligo

## Introduction

Cutaneous T-cell lymphomas (CTCLs) are extranodal non-Hodgkin lymphomas localized to the skin.^1^ Mycosis fungoides and Sezary syndrome (SS) are the most common types of CTCL, accounting for approximately two-thirds of all diagnosed cases of CTCL annually.^2^ Sezary syndrome, characterized by systemic dissemination of leukemic cells, classically presents as generalized erythroderma with lymphadenopathy but is notorious for its protean cutaneous manifestations.^1^^,^^2^ Diagnosis can be challenging, requiring the integration of clinical presentation, histopathology, immunopathology, and molecular evaluation for T-cell clonality.^1^ Treatment is stage-dependent and spans the spectrum of skin-directed to systemic therapies, the latter including retinoids, interferon, chemotherapy, and histone deacetylase inhibitors, as well as newer agents such as mogamulizumab (monoclonal antibody targeting C-C chemokine receptor 4 [CCR4]) and brentuximab vedotin (anti-CD30 monoclonal antibody-drug conjugate).^2^, ^3^, ^4^ CTCL-associated vitiligo-like leukoderma is a particularly rare manifestation, for which the optimal treatment regimen is unclear (Table I).^5^, ^6^, ^7^, ^8^, ^9^ Herein, we describe a patient with a striking example of this presentation, its associated challenges in diagnosis, and an excellent response to extracorporeal photopheresis (ECP) with mogamulizumab.

**Table I:** A summary of the reported cases of vitiligo-like leukoderma in setting of CTCL

First author (y)	Age, sex	Clinical description	Clinical diagnosis, stage	Treatment	Outcome
Alcalay, 1987^5^	62, Male	Erythroderma, lymphadenopathy, muscle wasting, depigmentation	Sezary syndrome	Topical and systemic steroids, chlorambucil	Stable disease
Bouloc, 2000^6^	83, Female	Erythroderma, pruritus, poikiloderma, lymphadenopathy, depigmented patches	Sezary syndrome	Topical and systemic steroids, topical mechlorethamine, systemic chlorambucil	Partial response
	65, Female	Erythroderma, pruritus, lymphadenopathy, depigmented patches	Sezary syndrome	Topical and systemic steroids, topical mechlorethamine, retinoids, PUVA, interferon-alfa2, methotrexate, chlorambucil, cytopheresis, total-body electron beam therapy.	Partial response
	65, Male	Erythroderma, depigmented patches	Erythrodermic MF	Topical and systemic steroids, topical mechlorethamine, PUVA, chlorambucil, methotrexate	Complete response
	63, Male	Psoriasiform plaques progressed to erythroderma, pruritus, lymphadenopathy, depigmented patches	Erythrodermic MF	Topical mechlorethamine	Complete response
Macheiner, 2003^7^	50, Male	Erythroderma, pruritus, lymphadenopathy, splenomegaly, symmetric polyarthritis	Sezary syndrome	Topical and systemic steroids, PUVA, localized radiation, vinblastine, cyclophosphamide, chlorambucil, ECP	Progression of disease
Knol, 2005^8^	71, Female	Erythroderma, pruritus, depigmented patches	Sezary syndrome	Interferon-alfa2 + PUVA, acitretin	Stable disease
Mirza, 2024^4^	62, Male	Erythroderma, hyperkeratosis of feet, lymphadenopathy, depigmentation with leukotrichia	Sezary syndrome T4 N3 B1 M0	Mogamulizumab	Partial response
Anderson, (current patient)	57, Female	Erythroderma, pruritus, lymphadenopathy, depigmented patches	Sezary Syndrome T4 N3 B1 M0	ECP Mogamulizumab	Partial response

*CTCL*, Cutaneous T-cell lymphoma; *ECP*, extracorporeal photopheresis; *MF*, mycosis fungoides; *PUVA*, psoralen + UVA phototherapy.

## Case presentation

**Fig 1:**
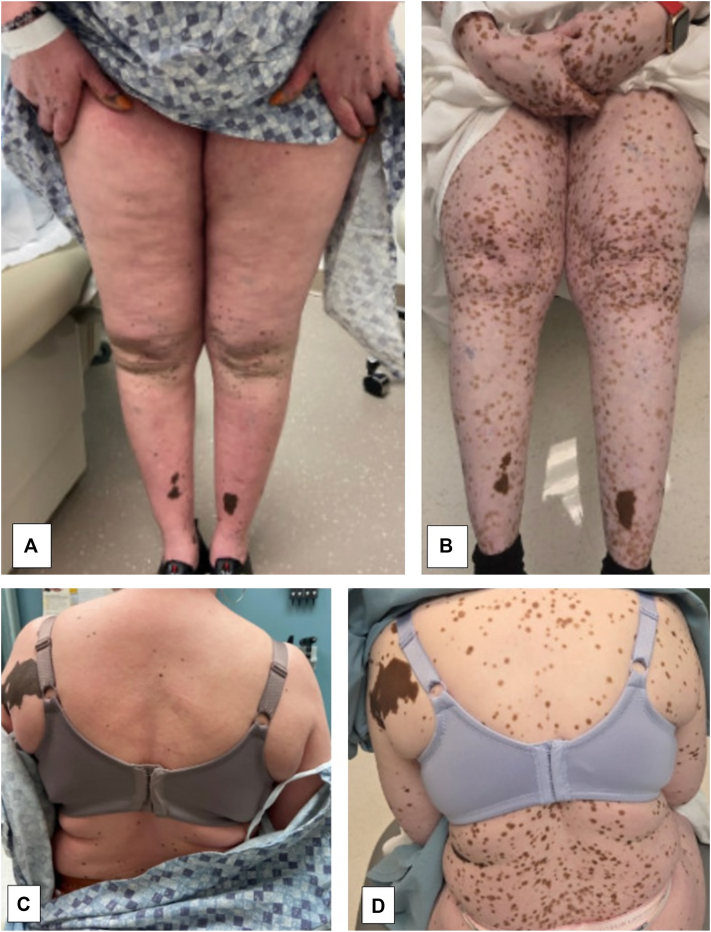
Pigmentation **(A** and **C)** while being treated with methotrexate and **(B** and **D)** while being treated with ECP and mogamulizumab. *ECP*, Extracorporeal photopheresis.

A 57-year-old Caribbean-American female presented with presumed vitiligo. She was referred to dermatology for rash, pruritus, and hypopigmentation, which developed following COVID-19 vaccination. The hypopigmentation began on the face and hands and subsequently spread diffusely, affecting over 80% of the body surface area, with some retention of perifollicular pigmentation (Fig 1). Additionally, a complete blood count demonstrated an absolute lymphocytosis initially felt to be reactive. Several initial skin biopsies showed postinflammatory hypopigmentation with spongiotic and psoriasiform dermatitis. She was treated with topical steroids, phototherapy, and methotrexate for presumed severe atopic dermatitis and concomitant vitiligo but witnessed no clinical improvement.

**Fig 2:**
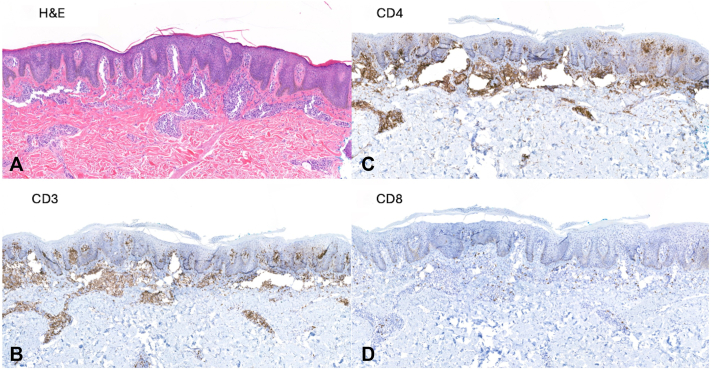
Skin biopsy from left shoulder depigmented patch, showing **(A)** psoriasiform epidermal hyperplasia with associated epidermotropic atypical lymphocytic infiltrate [H&E; 86×], comprised by **(B)** predominant T-lymphocytes [CD3; 73×], the majority of which are shown to represent **(C)** CD4-positive helper T-cells [CD4; 69×] with admixed **(D)** minority CD8-positive cytotoxic/suppressor T-cells [CD8; 79×].

**Fig 3:**
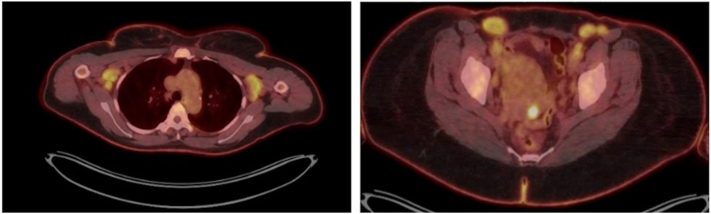
Initial whole-body positron emission tomography/computed tomography showing multiple fluorodeoxyglucose-avid enlarged bilateral axillary *(left)* and inguinal *(right)* lymph nodes, suggestive of lymphomatous involvement. The dominant right inguinal lymph node, measuring 3.4 × 2.3 cm, was subsequently biopsied, showing florid lymphomatous involvement (N3).

Given her minimal response to treatment, further evaluation of her lymphocytosis was undertaken. Laboratory evaluation revealed white blood count 14.2 k/mm3 (normal range: 3.7- 10.5) with absolute lymphocyte count of 7652 cells/mm3 (normal range: 875-3300), lactate dehydrogenase 439 U/L (normal range: 135-214), and human T-lymphotropic virus-type 1 and 2 antibody negative. Peripheral blood flow cytometry identified a monoclonal lymphoid population of CD4+, CD3+, CD5+, CD7 partial positive, and CD26 mostly negative cells, and T-cell gene rearrangement studies by polymerase chain reaction were positive, consistent with SS. The clonal population measured 4960 cells/mm3, qualifying as B2 staging. Subsequent cutaneous biopsies showed psoriasiform dermatitis with a monoclonal CD4-positive epidermotropic lymphoid proliferation (Fig 2). SOX10 immunohistochemical staining was absent within the epidermis, consistent with vitiligo in the background of CTCL. Positron emission tomography/computed tomography (PET/CT) imaging demonstrated multiple fluorodeoxyglucose (radioactive sugar tracer used in PET scans) (FDG)-avid enlarged axillary (largest: left axillary node 4.7 × 2.3 cm, standardized uptake value max 5.3) and inguinal lymph nodes (largest: right inguinal node 3.4 × 2.3 cm, standardized uptake value max 5.3) (Fig 3). A follow-up lymph node biopsy of the right inguinal node showed architectural effacement by a monoclonal CD4+, CD7-lymphoid proliferation, consistent with lymphomatous involvement. T-cell gene rearrangement studies on skin, blood, and lymph node all showed matching monoclonal peaks, overall consistent with a diagnosis of SS, stage IVA_2_ (T4N3bM0B2b).

She began monthly ECP treatments and mogamulizumab. After 3 cycles of therapy, there was improvement in the degree of erythroderma and pruritus, and her skin began slowly repigmenting. Interval peripheral blood flow cytometry showed complete resolution of her clonal lymphocytosis. Interim PET/CT showed decreasing size of her axillary and inguinal lymph nodes, with no new nodal growth. After completing 6 cycles of ECP + mogamulizumab, her pruritus remained resolved, as did her peripheral blood lymphocytosis. PET/CT showed quiescence of cutaneous disease but persistent FDG-avid bilateral axillary and inguinal lymphadenopathy. An axillary lymph node was rebiopsied, showing reactive lymphoid hyperplasia, consistent with dermatopathic lymphadenopathy. To date, she has continued combination therapy for over 1 year with an ongoing excellent response.

## Discussion

Although CTCL can manifest within a spectrum of pigmentation morphologies, few cases of CTCL presenting as vitiligo have been reported in the literature, as reviewed in Table I.^5^, ^6^, ^7^, ^8^, ^9^ These cases demonstrate the heterogeneity in both clinical presentation and applied therapies to patients with vitiligo-like leukoderma and highlight the absence of a consensus approach to this striking phenomenon.

Whether the initial depigmentation represents a true manifestation of early SS or authentic vitiligo as a precursor paraneoplastic epiphenomenon is challenging to definitively discern. Several mechanisms have been proposed to explain this immune-mediated depigmentation; vitiligo in the setting of CTCL is thought to result from destruction of epidermal melanocytes by tumoral lymphocytes, reactive T lymphocytes, or T-cell-activated B-cell-mediated autoantibodies.^7^ It has been hypothesized that since both CTCL and vitiligo involve abnormal immune activity, the dysfunctional T-cell activity in CTCL may predispose patients to melanocyte destruction.^7^^,^^9^ For example, patients with CTCL and vitiligo have been found to harbor subpopulations of CD8+ T cells reactive to MART-1 in their lesional tissue.^9^ The leading hypothesis proposes that malignant or reactive T-cells directly attack melanocytes after infiltrating the skin through pathways that normally recruit T-cells, utilizing cutaneous lymphocyte antigen and chemokine receptors such as CCR4.^5^^,^^9^^,^^10^

Our case and an additional case presented by Mirza et al showed benefit in repigmentation of leukodermic patches with the use of mogamulizumab, as seen in Fig 1.^5^ Mogamulizumab is a monoclonal antibody targeting CCR4, which is present on T-cells and consistently expressed by Sézary cells.^3^ Targeting CCR4 leads to antibody-dependent cellular cytotoxicity.^3^ In both cases, leukodermic patches were present prior to the initiation of treatment with mogamulizumab and improved after treatment.^5^ This suggests that mogamulizumab may treat both the cutaneous lymphoma and the vitiligo-like leukoderma. Of note, mogamulizumab has been associated with the onset of vitiligo after treatment initiation.^10^ While the pathogenesis is not entirely established, it is currently hypothesized that the loss of Treg cells leads to decreased immune tolerance and subsequently increased autoimmunity.^10^ Given our suggestion that mogamulizumab may be beneficial in improving dyspigmentation, more research is required to understand the interactions between mogamulizumab, the immune system, and melanocytes.

In conclusion, CTCL-induced vitiligo is rare and poorly understood. It is important for dermatologists to be aware of the various presentations of CTCL, including vitiligo, to limit delay in diagnosis. Further research is needed to better understand the mechanisms underlying the concomitant diagnoses of CTCL and associated vitiligo-like leukoderma and to optimize management of both the cutaneous lymphoma and skin dyspigmentation.
